# Imaging with the fluorogenic dye Basic Fuchsin reveals subcellular patterning and ecotype variation of lignification in *Brachypodium distachyon*

**DOI:** 10.1093/jxb/erv158

**Published:** 2015-04-28

**Authors:** Nikki Kapp, William J. Barnes, Tom L. Richard, Charles T. Anderson

**Affiliations:** ^1^Center for Lignocellulose Structure and Formation, The Pennsylvania State University, University Park, PA 16802, USA; ^2^Department of Biology, The Pennsylvania State University, University Park, PA 16802, USA; ^3^Department of Agricultural and Biological Engineering, The Pennsylvania State University, University Park, PA 16802, USA

**Keywords:** Acetyl bromide soluble lignin, Basic Fuchsin, *Brachypodium distachyon*, ecotype, flowering time, fluorescence imaging, lignin, Wiesner staining.

## Abstract

Lignin is a complex polyphenolic heteropolymer that is abundant in the secondary cell walls of plants and functions in growth and defence. It is also a major barrier to the deconstruction of plant biomass for bioenergy production, but the spatiotemporal details of how lignin is deposited in actively lignifying tissues and the precise relationships between wall lignification in different cell types and developmental events, such as flowering, are incompletely understood. Here, the lignin-detecting fluorogenic dye, Basic Fuchsin, was adapted to enable comparative fluorescence-based imaging of lignin in the basal internodes of three *Brachypodium distachyon* ecotypes that display divergent flowering times. It was found that the extent and intensity of Basic Fuchsin fluorescence increase over time in the Bd21-3 ecotype, that Basic Fuchsin staining is more widespread and intense in 4-week-old Bd21-3 and Adi-10 basal internodes than in Bd1-1 internodes, and that Basic Fuchsin staining reveals subcellular patterns of lignin in vascular and interfascicular fibre cell walls. Basic Fuchsin fluorescence did not correlate with lignin quantification by acetyl bromide analysis, indicating that whole-plant and subcellular lignin analyses provide distinct information about the extent and patterns of lignification in *B. distachyon*. Finally, it was found that flowering time correlated with a transient increase in total lignin, but did not correlate strongly with the patterning of stem lignification, suggesting that additional developmental pathways might regulate secondary wall formation in grasses. This study provides a new comparative tool for imaging lignin in plants and helps inform our views of how lignification proceeds in grasses.

## Highlight

Rapid visualization and relative quantification of lignin staining in stems of *Brachypodium distachyon* using the fluorogenic dye Basic Fuchsin illuminates spatio-temporal patterns and subcellular relationships of lignification in grasses.

## Introduction

A subset of the 10,000-species taxonomic family of grasses, otherwise known as Poaceae, provides the primary caloric intake of the world’s population, both by feeding livestock and as a direct source of food for human consumption. Sugarcane (*Saccharum* spp.) and maize (*Zea mays* L.) currently supply most of the world’s biofuels and other grasses have great potential as future renewable energy resources. In an effort to generate biofuels from sustainable feedstocks, attention has turned to lignocellulosic biomass, including crop residues and the non-edible plant parts such as grass stems and leaves (Margeot *et al.*, 2009).

A major constituent of lignocellulosic cell walls is lignin, a polymer composed of multiple types of phenylpropanoid subunits, which provides structural support and hydrophobicity for water transport, pathogen defence, and mechanical strength to vascular plants (Boerjan *et al.*, 2003). A primary obstacle to the saccharification of lignocellulosic biomass into sugar intermediates that can be fermented into biofuels lies in the recalcitrant nature of plant cell walls. During wall formation, polysaccharides are secreted or extruded into the apoplast and associate into complex interconnected networks which, in secondary walls, also include lignin (Cosgrove, 2005). Lignocellulosic biomass is recalcitrant to degradation by the hydrolytic enzymes involved in both rumen digestion and biomass saccharification (Jung *et al.*, 2012), and this recalcitrance has long been known to increase with lignin content (Patton and Gieseker, 1942; Van Soest, 1994). Despite the tremendous economic potential associated with increasing lignocellulosic biomass digestibility for both food and fuel, the fundamental basis of biomass recalcitrance remains poorly understood.

Current research to produce more economically viable lignocellulosic biomass feedstocks focuses on reducing lignin content or altering lignin structure in biomass to minimize the barrier lignin poses to cell wall degradation (Gomez *et al.*, 2008; Wilkerson *et al.*, 2014). Essential to this progress is a thorough understanding of the lignification process in proposed biofuel crops such as switchgrass (*Panicum virgatum*) and *Miscanthus×giganteus* (Somerville *et al.*, 2010). Biochemical methods for measuring total lignin provide key information about lignin content, but typically require tissue disruption and pretreatment to separate lignin from the cell wall matrix (Hatfield and Fukushima, 2005). As a complementary approach to biochemical quantification, visualizing lignin across different tissue types is very useful for qualitatively assessing the spatial distribution of lignin in a sample. Lignin autofluorescence has been used to determine its localization in plant tissues (De Micco and Aronne, 2007), but the autofluorescence signals of lignin can sometimes be confounded by the presence of additional autofluoresent compounds in the plant. Dyes used specifically to visualize lignin include Wiesner reagent (Vallet *et al.*, 1996), Mäule (Gibbs, 1958; Meshitsuka and Nakano, 1978, 1979), chlorine-sulphite (Akin and Burdick, 1981), and Basic Fuchsin (Dharmawardhana *et al.*, 1992). However, there is currently no reported quantitative, specific, and rapid method for spatially localizing lignin in intact plant tissues.

Lignin deposition follows distinct spatial-temporal patterns. Generally, stems contain the highest concentration of lignin in vascular plants and all mature stem tissue is lignified to some extent in grasses (Hatfield *et al.*, 2009). Lignin content in crops depends on factors such as growth stage, genotype, morphology, and environmental conditions (Frei, 2013). Within stem tissue, lignin has been found to be most abundant towards the base of the stem where the tissue is most mature (Chen *et al.*, 2002; Zhang *et al.*, 2014). Temporally, lignin content (as well as overall cell wall content) accumulates throughout the development of vascular plants, resulting in increased recalcitrance in mature biomass. The spatial distribution of lignin varies by cell type with a higher abundance in xylem vessels and tracheids, which function in transporting water, and structural fibres, which maintain plant structural integrity (Matos *et al.*, 2013).

*Brachypodium distachyon* (L.) P. Beauv is an annual, temperate, C3-photosynthesizing grass native to the Middle East and is a relative of agronomically valuable cereal crops including wheat, barley, oats, maize, rice, rye, sorghum, and millet (Kellogg, 2001; The International Brachypodium Initiative, 2010). With a small diploid genome (~300 Mbp), small stature, short generation time (8–12 weeks), and simple growth conditions, *Brachypodium* has emerged as a useful model species for monocot grasses (Draper *et al.*, 2001). Current models also used for grass crop research include rice (*Oryza sativa*) and maize (*Zea mays*); however, these crop species have large genomes and labour-intensive growth requirements, making rapid laboratory-based experimentation a challenge. Pushed by the need for a suitable model for functional studies of grasses (notably for crop, forage, and biofuels), the *B. distachyon* research community has begun to form a collaborative consortium akin to that for *Arabidopsis thaliana* (L.) Heynh, which has served as a model dicot species for over three decades. Advances in publically available *B. distachyon* resources include an assembled genome, a growing number of available T-DNA lines, and protocols for efficient crossing and transformation (Brkljacic *et al.*, 2011).

In this work, the fluorogenic dye, Basic Fuchsin, was used as a probe to measure lignification in transverse, basal, internode stem sections of *B. distachyon* and assess differences in lignification for three *B. distachyon* ecotypes that vary in flowering time. Quantitative analysis of Basic Fuchsin fluorescence was compared with Wiesner staining and biochemical measurement of lignin in whole aerial plant cell walls as measured by the acetyl bromide method. It was found that Basic Fuchsin fluorescence is a rapid and informative method for determining the spatial abundance of lignin in grass cell walls, revealing subcellular details of lignification patterns. It was also found that Basic Fuchsin fluorescence increases to varying degrees over developmental time in the *B. distachyon* ecotypes Bd21-3, Bd1-1, and Adi-10, reflecting differences in lignification between these flowering time variants.

## Materials and methods

### Plant material

*B. distachyon* ecotypes Bd21-3, Bd1-1, and Adi-10 (kindly provided by Dr John Vogel, Joint Genome Institute, Walnut Creek, CA, USA) were used throughout this study. These ecotypes were selected based on their natural variation in flowering time (Schwartz *et al.*, 2010; Tyler *et al.*, 2014). *B. distachyon p*-coumaryl-CoA:monolignol transferase overexpressor line (*BdPMT OX*) and Bd21-3 seeds were kindly provided by Deborah Petrik, Illinois State University, Normal, IL, USA. Seeds were sterilized following lemma removal, sown in soil (1:1 v/v Metro Mix 360 and Fafard C2 mix) in 6.4cm diameter square pots, and vernalized for 2 weeks at 4 °C in the dark. Pots were ordered in 8×4 arrays in flats, alternating ecotypes with each subsequent pot to ensure consistent growth conditions across all ecotypes. Following vernalization, plants were grown in a temperature-controlled growth chamber (22 °C, 20h light; Percival PGC-9/2).

*Arabidopsis thaliana* Columbia ecotype*, fah1-2*, and *ref3-2* seeds (ABRC) were surface-sterilized by soaking in 500 μl of 30% bleach+0.1% sodium dodecyl sulphate for 20min in 1.7ml microcentrifuge tubes, vortexing every 10min. Seeds were then washed four times with sterile water in a biohood and suspended in 500 μl of sterile 0.15% agar in water. Sterilized seeds were stored at 4 °C in the dark for 3 d (seeds can be sown on plates between 3 d and 10 d after sterilization). Seeds were sown on sterile plates containing 2.2g l^–1^ Murashige and Skoog salts (Caisson Labs), 0.6g l^–1^ 2-(*N*-morpholino)ethanesulphonic acid (Research), 8g l^–1^ agar-agar (Research Organics), and 1% sucrose (Sigma) using a 200 μl pipette with cut-off tips. Two rows of seeds were plated on each plate with ten seeds per row and left to dry in the biohood for 45min. Plates were taped closed with micropore tape (3M) to prevent contamination and placed vertically in a Percival CU-36L5 growth chamber at 22 °C and with a 24h light period for 5 d. At 10 d after germination, seedlings were transferred to soil with MiracleGro and grown in a temperature-controlled growth chamber (22 °C, 16h light) for 5 weeks.

### Growth stage

Flowering was assessed for a population of 30 *B. distachyon* plants of each ecotype by scoring growth stage every 3 d until 89 d after germination (DAG) based on the BBCH scale established for *B. distachyon* (Hong *et al.*, 2011). Heading was considered to be the beginning of flowering and any growth stage of 51 (beginning of heading, tip of inflorescence emerged from sheath, approximately 26.5 d for Bd21-3 growth) or greater was considered as flowering.

### Section preparation

Five-week-old *A. thaliana* Col, *fah1*-2, and *ref3*-2 basal-primary stems were hand-sectioned with a razor blade. Five plants of each *B. distachyon* ecotype were randomly selected for stem sectioning at 28 d (day zero corresponds to the day pots were transferred to the growth chamber). Approximately 5mm of basal internode tissue of each plant was dissected with a new razor blade and embedded in a 10×10×5mm plastic mould filled with Shandon^TM^ Cryomatrix^TM^ cooled using liquid nitrogen. Embedded samples were stored overnight at –80 °C before sectioning. The remaining aerial plant mass for each plant sample was freeze-dried for downstream cell wall isolation and acetyl bromide soluble lignin quantification. Cross-sections of 25 μm thickness were cut on a Leica cryostat at –25 °C with a soft tissue blade and transferred on to Thermo Superfrost positively charged slides with three sections per slide for staining and microscopy. For each sample, one slide was used for Wiesner staining and another was used for Basic Fuchsin staining. Stem sections were saponified by adding 15 μl of 25% 1.5M NaOH (or MilliQ water for controls) to the surface of each section, and incubating at room temperature for 30min (Kim and Carpita, 1992). NaOH was removed by pipetting or wicked off with laboratory tissue and sections were washed three times with water before Basic Fuchsin staining.

### Tissue staining

Slides were prepared for staining by solubilizing the Cryomatrix around each section with a drop of water and wicking away the Cryomatrix and water with a laboratory tissue. Each section was encircled with a liquid blocking Pap Pen (Sigma). For Basic Fuchsin staining, *A. thaliana* and *B. distachyon* stem sections were incubated in 15 μl of 0.001% or 0.01% Basic Fuchsin (Sigma 857343, dye content ≥88%), respectively, in water for 5min, washed twice with 50% glycerol (v/v) (10min per wash), and mounted in 50% glycerol. Basic Fuchsin-stained tissues were imaged on a Zeiss Cell Observer SD spinning disc confocal microscope with 20×0.50 NA and 63×1.4 NA objectives under 561nm excitation and 593/40nm emission and a Photometrics QuantEM 512SC EMCCD camera. The MosaiX tool in Zeiss AxioVision 4.8 was used to capture whole stem cross-sectional regions. Wiesner reagent (phloroglucinol in hydrochloric acid) staining was used for a qualitative assessment of lignin distribution in stem sections. Two per cent (w/v) phloroglucinol (Sigma-Aldrich) in 95% ethanol was mixed 2:1 (v/v) with concentrated hydrochloric acid. Before staining, 15 μl of 70% ethanol was transferred to each section for 10min to remove chlorophyll. Each section was rehydrated with 15 μl dH2O, which was wicked off the slide with a laboratory tissue before staining with 15 μl of 2% Wiesner reagent for 5min. Residual stain was removed with a tissue before mounting slides in 50% (v/v) glycerol. Wiesner-stained sections were immediately imaged under brightfield with a 10× objective on an Olympus BX51 using Progress Capture 2.7.7 imaging software.

### Basic Fuchsin fluorescence quantification

After imaging, the fluorescent area above background for each Basic Fuchsin-stained section was selected in ImageJ (NIH) using the default auto-threshold plugin with ‘ignore black’ and ‘ignore white’ boxes checked to account for any under- or over-exposed pixels (http://fiji.sc/Auto_Threshold). Because this algorithm converts images to 8-bit depth, the thresholded area was then selected, saved as an ROI, and re-applied to the original 16-bit image. The raw integrated density (sum of all pixel intensities) of the thresholded area was then measured. This value was normalized to the whole stem area as measured from one stem cross-section for each plant by tracing the section perimeter in ImageJ. The area-normalized Basic Fuchsin fluorescence intensity values represent the amount of Basic Fuchsin staining for each section and serve as a quantitative measurement of staining within that section.

### Cell wall preparation

Freeze-dried, whole aerial plant material was coarsely ground using a Wiley mini-mill and finely ground in a cryomill using the automated pre-cooling cryo setting (~3min, 5 Hz) followed by non-cryo (5min, 30 Hz). Starch-free cell wall preparations were made based on the procedures of Hatfield *et al.* (2009). Seventy milligrams of cryomilled plant material of each sample was weighed out into 2ml Sarstedt tubes to which 1.5ml of 70% ethanol was added, vortexed, and centrifuged in an Eppendorf 5424 micro-centrifuge (10 000rpm for 10min) to pellet the insoluble residue. The supernatant was decanted and 1.5ml of chloroform/methanol (1:1 v/v) was added. The resuspended pellet was centrifuged at 10 000rpm for 10min, supernatant decanted, and the pellet resuspended in 500 μl of acetone. The solvent was evaporated with an air stream at 35 °C until dry (samples can be stored after this step for later use) and resuspended in 1.5ml of 0.1M sodium acetate buffer (pH 5.0) to initiate starch removal. Samples were heated for 20min at 80 °C in a heating block and cooled on ice. A solution of 35 μl of 0.01% sodium azide (NaN3), 35 μl amylase (50 μg ml^–1^ H_2_O; from *Bacillus* species, Sigma); 17 μl pullulanase (17.8 units from *Bacillus acidopullulyticus*, Sigma) was added to the pellet. The suspension was vortexed thoroughly and incubated overnight at 37 °C on a shaker, lying horizontally to improve mixing. The next morning, the suspension was heated at 100 °C for 10min to terminate starch digestion, centrifuged (10 000rpm for 10min), the supernatant discarded, and the pellet washed three times with dH_2_O (vortexing, centrifuging, and decanting each time). The pellet was resuspended in 500 μl of acetone and evaporated with a stream of air in a 35 °C heating block until dry. To remove any residual starch, an additional overnight treatment with 90% DMSO (Sigma-Aldrich) at room temperature with occasional vortexing was performed, followed by one 90% DMSO wash step, and six 70% ethanol wash steps before re-suspending in acetone and air drying the de-starched alcohol-insoluble residue (Fry, 1988). The absence of starch was verified by staining with 5% Lugol’s iodine.

### Acetyl bromide soluble lignin (ABSL) measurements

The acetyl bromide method used for determining lignin content is based on that of the microscale method using cuvettes described by Chang *et al.* (2008). Five milligrams of prepared AIR was weighed. The exact weight transferred was recorded for each sample for more accurate per cent ABSL calculation. Weighed cell wall isolate was transferred to 7ml glass screw-cap vials (Sigma) containing 1ml of 25% (v/v) acetyl bromide (Sigma) in glacial acetic acid and incubated for 1h in a water bath in a secondary container set to 70 °C, with gentle shaking every 10min. One control of only 25% AcBr was prepared to use as a blank for spectroscopy. After incubation, samples were cooled on ice. After cooling, 5ml of glacial acetic acid was added to each vial, vortexed thoroughly, and samples were left to sit overnight in the fume hood to allow for particulates to settle. Absorbance was measured at 280nm on a Nanodrop 2000C using a quartz cuvette after adding 500 μl of glacial acetic acid, 150 μl of the acetyl bromide reaction mixture, 200 μl 1.5M NaOH, and then 150 μl of fresh 0.5M hydroxylamine hydrochloride in water to the cuvette, followed by thorough mixing in the cuvette via pipetting. The cuvette was washed with 1ml glacial acetic acid between each sample. The spectrophotometer was blanked with the control solution. The extinction coefficient used for *Brachypodium* is 18.126g^–1^cm^–1^ based on the average values determined for C3 grasses (Fukushima and Hatfield, 2004). Cell wall preparations were analysed in triplicate and data were compiled according to plant accession and age. Per cent ABSL was calculated based on Beer’s Law:

$$\begin{array}{l} \%\text{ ABSL}=\left[A^{\text{28}0}/\varepsilon *\text{ pathlength }\left(\text{cm}\right)\right]\\ \;\times \left[\text{dilution factor}/\text{mass }\left(\text{mg}\right)\right]\times \text{1}00\% \end{array}$$

## Results and discussion

### Basic Fuchsin staining is indicative of the presence of lignin in plant cell walls

Wiesner reagent (phloroglucinol in HCl) has been used as a standard stain for qualitatively assessing the presence of lignin in plant material. Phloroglucinol is a phenol derivative, which, under acidic conditions, has been shown to stain O-4-linked coniferyl and sinapyl aldehydes in lignifying cell walls (Pomar *et al.*, 2002). However, no method has been reported accurately to quantify the relative amounts of lignin present in different anatomical locations based on the pigmentation of plant tissue by the Wiesner reagent. Despite efforts to quantify stains like Wiesner, because they are visualized as colour brightfield images where pixel intensities arise from multiple channels, selecting relevant pixel values in an unbiased way remains problematic. Basic Fuchsin is a triaminotriphenylmethane dye widely used in the textile industry as a colouring agent and in cell biology as a stain for collagen, muscle, and mitochondria (Pathrose *et al.*, 2014). Basic Fuchsin has also been used in a variety of plant species as a stain for lignified cell walls (Dharmawardhana *et al.*, 1992, 1995; Caño-Delgado *et al.*, 2000; Moller *et al.*, 2005; Wagner *et al.*, 2009; Smith *et al.*, 2013; Rocha *et al.*, 2014). Although the specific targets and reaction mechanism of this dye have not been characterized, Basic Fuchsin staining intensity qualitatively correlates with lignin abundance in wild-type and mutant *Pinus radiata* cell walls (Wagner *et al.*, 2009), but Basic Fuchsin staining has not previously been reported as a method quantitatively to compare the relative amounts of lignin present within plant tissues.

**Fig. 1.**
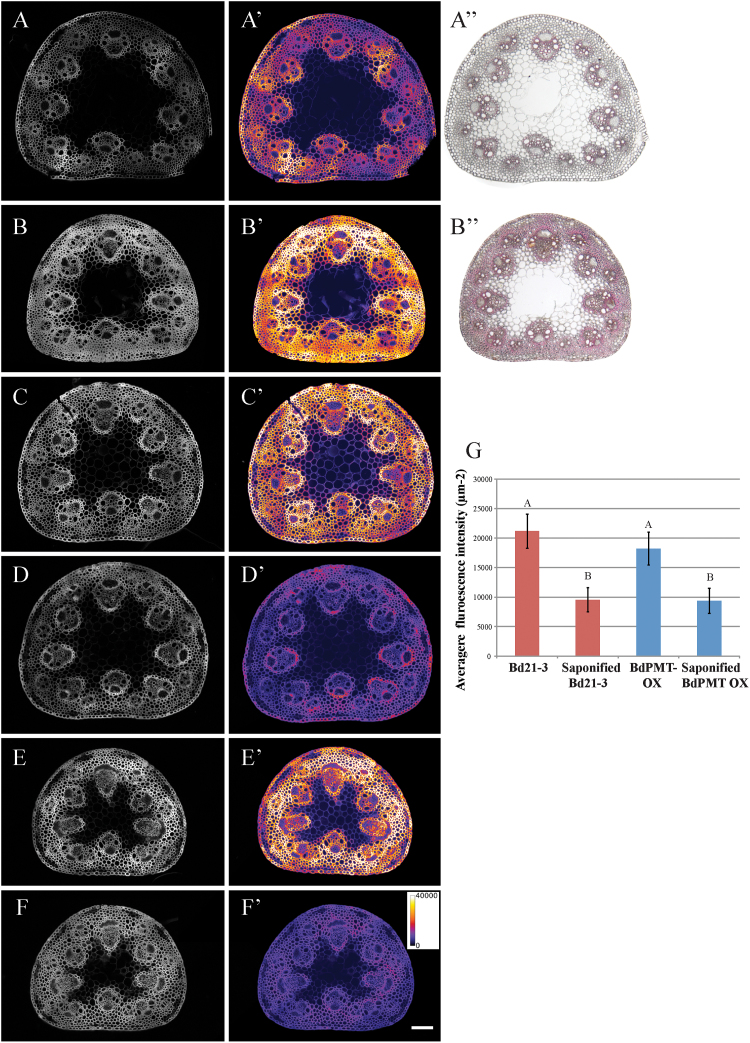
Basic Fuchsin staining increases over the course of *B. distachyon* development. Basic Fuchsin fluorescence intensity μm^–2^ increases 2.04-fold and Wiesner staining increases in *B. distachyon* ecotype Bd21-3 from 4 weeks (A, A’, A’’) to 12 weeks (B, B’, B’’). Average pixel intensity above threshold μm^–2^ is 14 713 (AFU) for (A) and 34 389 (AFU) for (B). Basic Fuchsin fluorescence intensity of 4-week-old Bd21-3 (C) and *BdPMT OX* (E) plants decreases after saponification (D, F) by approximately 2-fold (G). Greyscale representations of Basic Fuchsin fluorescence (A–F), Basic Fuchsin fluorescence with a pseudo-colour look-up table applied (A’–F’) where the intensity profile shows pixel intensity values for different colours, and Wiesner staining (A’’, B’’). Scale bar=100 μm. Error bars in (G) show standard error; values with different superscript letters indicate significant differences between each ecotype pair measured by one-way ANOVA and post-hoc test, *P* <0.05.

To test whether Basic Fuchsin staining changes over time to reflect developmental increases in stem lignin content, 4-week-old and 12-week-old *B. distachyon* ecotype Bd21-3 basal stem sections were analysed using Basic Fuchsin staining and the observed patterns of fluorescence were compared with those observed after staining with the Wiesner reagent (Fig. 1). The basal internode was of interest in this study because it represents the oldest stem tissue and is expected to accumulate lignin throughout development. Qualitative observation of lignin staining indicated that both Basic Fuchsin fluorescence and Wiesner staining were more widespread and pronounced in 12-week-old stem sections (Fig. 1). Quantitative measurement indicated that Basic Fuchsin fluorescence intensity μm^–2^ was 2.34-fold higher in 12-week-old Bd21-3 stems. It was observed that this increase arose predominantly from increased staining in interfascicular regions, supporting the well-documented spatio-temporal pattern of lignification in grasses such as *B. distachyon* throughout maturation (Matos *et al.*, 2013). By calculating the ratio of thresholded stem area to total stem area, it was possible to quantify the relative lignified area within a stem section. This number was 4.25-fold higher in 12-week-old Bd21-3 stems than in 4-week-old stems, suggesting that the increase in fluorescence intensity μm^–2^ can be attributed to an increase in Basic Fuchsin fluorescence intensity in previously lignified tissues as well as to an increase in lignified area in more mature tissue (Fig. 1). Basic Fuchsin fluorescence and Wiesner staining was also observed in the epidermis of 12-week-old Bd21-3 stems, possibly due to the presence of cutin or suberin which can also fluoresce when stained with Basic Fuchsin (Kraus *et al.*, 1998). Overall, the similar staining pattern between Basic Fuchsin and the Wiesner reagent supports the interpretation that Basic Fuchsin fluorescence intensity correlates with the amount and location of lignin in secondary cell walls.

### Basic Fuchsin is likely to stain lignin and other compounds such as hydroxycinnamic acids

In addition to the three canonical types of lignin (H, G, and S) that arise from *p*-coumaryl, coniferyl, and sinapyl alcohol, respectively, grass walls contain a high proportion of the hydroxycinnamates ferulic acid (FA) and *p*-coumaric acid (*p*CA) compared with non-grasses (Vogel, 2008). Despite the affinity of Basic Fuchsin to lignified tissues and cell walls, the specific target of the dye remains elusive. To investigate the affinity of Basic Fuchsin for different lignin compositions, staining was quantified in *BdPMT OX*, which displays a 3-fold higher *p*CA acylation of lignin but overall a lower lignin content compared with wild-type Bd21-3 (Petrik *et al.*, 2014). The Basic Fuchsin fluorescence intensity detected in 4-week-old *BdPMT OX* basal stem sections was approximately 86% of the intensity detected in Bd21-3 (Fig. 1C, E, G). This value is consistent with the ratio (81%) of the reported sum (151mg g^–1^) of Klason lignin (132mg g^–1^) and the hydroxycinnamates *p*CA+FA (19mg g^–1^) detected in *BdPMT OX* stems compared with Bd21-3 stems (186mg g^–1^ total; 171mg g^–1^ Klason lignin; 15mg g^–1^ *p*CA+FA) (Petrik *et al.*, 2014).

To investigate the contribution of *p*CA and FA to Basic Fuchsin staining, 4-week-old Bd21-3 and *BdPMT OX* basal stem sections were subjected to saponification to de-esterify hydroxycinammic acids before staining with Basic Fuchsin. Saponified, stained Bd21-3 sections had approximately half of the Basic Fuchsin fluorescence intensity μm^–2^ [9548±2046 (SE) AFU μm^–2^] compared with non-saponified control sections [21186±2897 (SE) AFU μm^–2^; *n*=14 sections from five plants per treatment; Fig. 1]. There was similarly a 2-fold decrease in *BdPMT OX* fluorescence intensity after saponification (Fig. 1G). The largest difference in fluorescence intensity before and after saponification was observed in the epidermis (see Supplementary Fig. S1 at *JXB* online) and is most likely due to the staining of cutin which is rich in esterified fatty acids (Nawrath, 2002). These data indicate that Basic Fuchsin has a high affinity for hydroxycinammates in the *B. distachyon* cell wall; although *p*CA can be linked to lignin, FA is almost exclusively linked to xylans in grass cell walls (Wilkerson *et al.*, 2014), which should be taken into account when interpreting the results of Basic Fuchsin staining experiments.

To test whether saponification was removing these grass-specific compounds and not simply extracting lignin, Basic Fuchsin staining before and after saponification was investigated in *A. thaliana* Col, which lacks the high levels of *p*CA and FA found in grasses. No significant change in Basic Fuchsin fluorescence intensity was observed after saponification (see Supplementary Fig. S1 at *JXB* online), indicating that, in *A. thaliana* stems, the warm alkali treatment used for saponification does not remove a significant portion of lignin from the sections.

Basic Fuchsin specificity was also investigated in stems of the previously characterized *fah1-2* and *ref3-2 A. thaliana* mutants, which show defects in lignin composition and abundance, respectively (Ruegger and Chapple, 2001). The *fah1-2* mutant has no detectable level of S lignin, but the amount of G lignin present in *fah1-2* alone accounts for, on average, 84.5% of the total lignin detected in wild-type tissue, with notable variation in measured lignin composition (Ruegger and Chapple, 2001). The level of Basic Fuchsin fluorescence detected in the *fah1-2* sections is slightly lower, but not significantly different, from that of Col sections (see Supplementary Fig. S2 at *JXB* online), indicating that in Col and *fah1-2* plants, a similar amount of total lignin coincides with a similar Basic Fuchsin fluorescence intensity in stained tissues despite dramatic differences in lignin composition. *ref3-2* plants have approximately one-quarter of the total amount of lignin and a similar S:G ratio as the wild type (Ruegger and Chapple, 2001). *ref3-2* sections were observed to have roughly two-thirds of the Basic Fuchsin fluorescence intensity as Col (see Supplementary Fig. S2 at *JXB* online). Together, these data indicate that Basic Fuchsin is sensitive to the relative amount of lignin between samples, stains hydroxycinnamates, and may be insensitive to lignin composition.

### *Brachypodium* ecotypes vary in spatiotemporal lignification patterns and flowering time

**Fig. 2.**
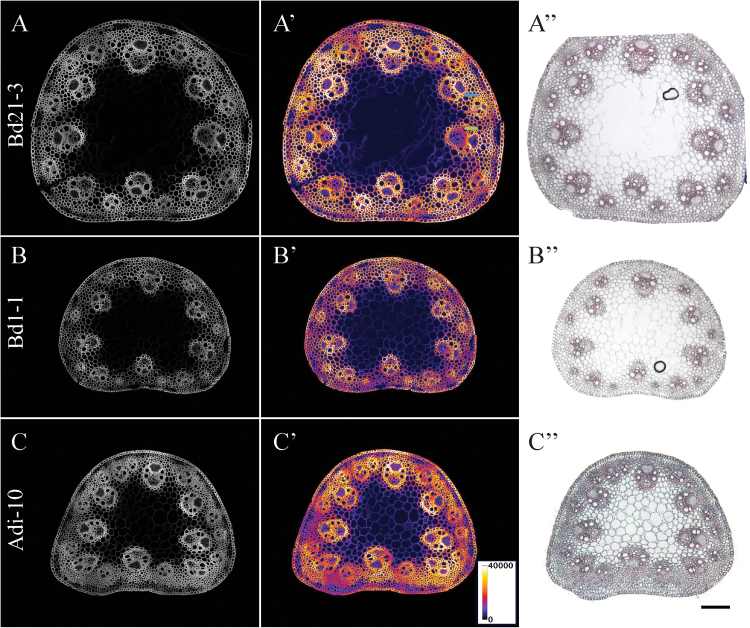
Basic Fuchsin fluorescence varies across *B. distachyon* ecotypes. Basic Fuchsin fluorescence (A, B, C), Basic Fuchsin fluorescence with a pseudo-colour look-up table applied (A’, B’, C’), and Wiesner staining (A’’, B’’, C’’) of base internode stem sections (25 μm thickness) from *B. distachyon* ecotypes Bd21-3 (A, A’), Bd1-1 (B, B’), and Adi-10 (C, C’) sampled at 4 weeks of growth. See Table 1 for quantification of staining intensities for the three ecotypes. Scale bar=100 μm.

*B. distachyon* ecotypes Bd1-1 and Adi-10 are both late-flowering compared with the ecotype Bd21-3, which displays synchronous growth and early flowering and is therefore often used as a control ecotype in *B. distachyon* studies (Vogel *et al.*, 2009; Schwartz *et al.*, 2010). Per cent heading was scored at 4 weeks for sets of Bd21-3, Bd1-1, and Adi-10 plants (Table 1; see Supplementary Table S1 at *JXB* online) to look for any correlation between flowering and lignification. In agreement with previous studies (Vogel *et al.*, 2009; Schwartz *et al.*, 2010), 90% of Bd21-3 plants had flowered by 4 weeks of growth, whereas 30% of Bd1-1 plants and 0% of Adi-10 plants had flowered (see Supplementary Fig. S3 at *JXB* online). Although Bd1-1 and Adi-10 were both late to flower, these ecotypes displayed different growth morphologies (see Supplementary Fig. S4 at *JXB* online) as well as different basal internode Basic Fuchsin staining intensity and patterning (Fig. 2).

**Table 1.** Basic Fuchsin fluorescence intensity is significantly greater at 4 weeks in Adi-10 and Bd21-3 than in Bd1-1 (*n*=15 sections from five plants per ecotype); whole aerial tissue %ABSL (cell wall dry weight) significantly differs between Bd21-3 and Bd1-1 at 4 weeks Values with different letters indicate significant differences between each ecotype pair measured by one-way ANOVA and post-hoc test, *P* <0.05.

Ecotype	% Flowered	Fluorescence intensityμm^–2^ (AFU)	Average stem area(μm^2^)	Thresholded area/Stem area	%ABSL
Bd21-3	90	14713±702 a	652977±49739 a	0.28±.01 a	22.70±1.56 a
Bd1-1	30	4728±1365 b	451788±36176 b	0.11±0.02 b	17.44±0.71 b
Adi-10	0	15866±1235 a	695334±61312 a	0.27±0.02 a	19.12±2.35 ab

Basic Fuchsin fluorescence was imaged and quantified in basal internode stem sections from 4-week-old Bd21-3, Bd1-1, and Adi-10 plants (Fig. 2; Table 1). Basic Fuchsin fluorescence intensity μm^–2^ in 4-week-old stems was significantly higher in Bd21-3 and Adi-10 than in Bd1-1. Overall, Bd1-1 plants were morphologically similar to Bd21-3 plants throughout development and both ecotypes were morphologically distinct from Adi-10 plants (see Supplementary Fig. S4 at *JXB* online), except that, at 4 weeks of growth Adi-10 plants looked nearly identical to Bd21-3 plants although they had not yet flowered. Basic Fuchsin and Wiesner staining was extensive and intense in both vascular bundles and interfascicular tissue in Bd21-3 and Adi-10 stems at 4 weeks, whereas staining was less intense in both vascular bundles and interfascicular tissue in Bd1-1 stems (Fig. 2). These results suggest that increasing lignin deposition in the stem might be more dependent on plant vegetative morphology than on developmental cues such as flowering.

### Quantification of Basic Fuchsin staining provides spatial information on lignification that is complementary to whole-plant ABSL measurements

**Fig. 3.**
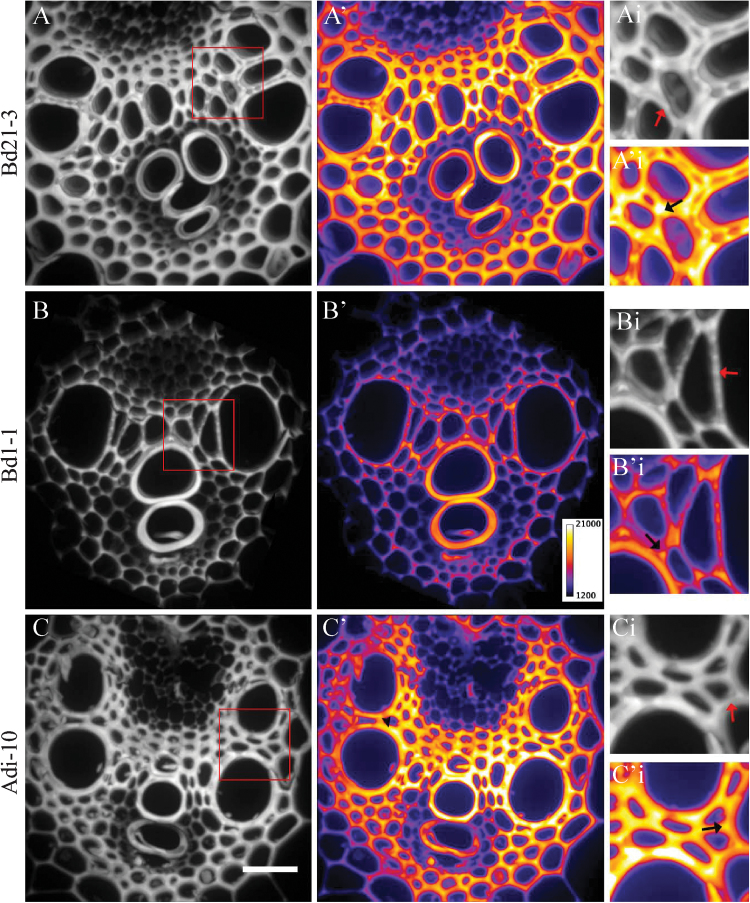
High-magnification imaging of Basic Fuchsin staining. Basic Fuchsin staining reveals subcellular maxima of fluorescence intensity in the middle lamellae and cell corners and lattice-like patterning in the walls of vessel elements of *B. distachyon* ecotypes Bd21-3 (A, A’, A’’), Bd1-1 (B, B’, B’’), and Adi-10 (C’, C’, C’’) sampled at 4 weeks of growth in grey-scale (A, B, C) and with a pseudo colour look-up table applied (A’, B’, C’). Insets show magnification of area outlined in red in A, B, and C, showing high-intensity staining at middle lamellae (Ai, Bi, Ci; red arrows) and cell corners (A’i, B’i, C’I; black arrows). Scale bar=20 μm.

At the subcellular level, Basic Fuchsin fluorescence was concentrated in the middle lamellae and cell corners in all three *B. distachyon* ecotypes (Fig. 3) where lignin polymerization is thought to initiate (Donaldson, 2001). More widespread Basic Fuchsin fluorescence was observed in Bd21-3 and Adi-10 vessel element and fibre cell walls than in Bd1-1 walls (Fig. 3). These results suggest that the differences in total Basic Fuchsin fluorescence between these ecotypes (Table 1) might arise both from differences in the number of cells within the stem that are lignified at a given developmental stage and the extent of lignin deposition within individual cell walls. Lattice-like gaps were also observed in Basic Fuchsin fluorescence adjacent to vessel elements in Bd21-3 stems that probably reflect the presence of non-lignified pits in these walls (Fig. 3).

To compare our imaging-based analysis of lignin distribution to larger-scale measurements of lignin content, ABSL was biochemically quantified using purified cell wall material from whole aerial *B. distachyon* tissues. The extinction coefficient (18.126g^–1^Lcm^–1^) used to calculate percentage ABSL in cell wall dry weight was an average of coefficients determined for several C3 grasses (Fukushima and Hatfield, 2004), since a coefficient specific for *B. distachyon* remains to be calculated. Rancour *et al.* (2012) found ABSL content to vary more by organ type than by developmental stage in *B. distachyon* (Bd21-3). In general, there was a lower percentage of ABSL at 2 weeks after germination for each ecotype, which increased after this time despite variability in the percentage ABSL over time within each ecotype and between ecotypes at 4, 6, 8, 10, and 12 weeks after germination (see Supplementary Fig. S5 at *JXB* online). This variability within each ecotype from week to week may be a result of altered ratios of tissue types contributing to aerial biomass with different total lignin contents as plants grew and developed. However, unlike Basic Fuchsin fluorescence intensity μm^–2^ within basal-internode stem sections, the changes in percentage ABSL over time were modest and more consistent from 2–12 weeks (see Supplementary Fig. S5 at *JXB* online). Notably, a spike in per cent ABSL occurred following the time point when plants for each ecotype began to reach the heading stage of development. For the Bd21-3 and Bd1-1 ecotypes, most plants that went on to head had done so at approximately 3 weeks and 5 weeks after germination, respectively, and percentage ABSL levels greatly increased at the subsequent time points for which measurements were conducted, i.e. at 4 weeks for Bd21-3 and 6 weeks for Bd1-1 (see Supplementary Figs S3 and S5 at *JXB* online). Adi-10 plants flowered much later than the other two ecotypes, beginning to head around 4.5 weeks after germination and, in this ecotype, the percentage ABSL increased and remained elevated at 6 to 12 weeks after germination, with the exception of a lower per cent ABSL level at 10 weeks (see Supplementary Figs S3 and S5 at *JXB* online). By contrast, a strong correlation between flowering time and Basic Fuchsin fluorescence intensity in basal internode stem sections was not observed, with the exception that an initial spike in intensity was evident for Bd21-3 at 4 weeks after germination (see Supplementary Fig. S6 at *JXB* online). Adi-10 displayed a consistent increase in the percentage of plants heading, percentage ABSL, and Basic Fuchsin fluorescence intensity over time, demonstrating how Basic Fuchsin staining is correlated with lignification on a spatio-temporal scale for this ecotype. Our results suggest that Basic Fuchsin staining in stem cross-sections reveals tissue- and cellular-level intricacies of the developmental lignification process that are not captured by biochemical quantification methods such as ABSL, which require cell wall disruption and extraction.

## Conclusions

Quantifying fluorescence intensity by imaging the fluorogenic dye Basic Fuchsin provides a rapid estimation of the concentrations of lignins and other hydrophobic compounds in plant tissue sections. Staining basal internode cross-sections with Basic Fuchsin produced a similar staining pattern as staining with Wiesner reagent (Figs 1, 2), which is known to bind to O-4-linked coniferyl and sinapyl aldehydes in lignifying cell walls (Pomar *et al.*, 2002). Although the precise binding mechanism of Basic Fuchsin with lignin is unknown, this dye has long been used as a test for the presence of aldehydes (Schiff’s tests; Tobie, 1942). Further analyses will be required to determine the precise mechanism by which Basic Fuchsin interacts with lignin to become fluorescent.

The use of Basic Fuchsin for quantitative imaging in *B. distachyon* and *A. thaliana* basal stem sections has been demonstrated here. Despite the finding that Basic Fuchsin does not exclusively stain lignin, the differences that were detected in Basic Fuchsin staining intensities and patterns at the tissue and cellular levels, compared with whole aerial plant ABSL, have the potential to be recapitulated in the developmental lignification patterns of bioenergy crop species. Finally, the fact that *B. distachyon* Adi-10 plants display relatively extensive lignification at 4 weeks, despite not having flowered, indicates that further investigation is required to determine the links between flowering time, lignification, and biomass recalcitrance.

## Supplementary data

Supplementary data can be found at *JXB* online.

Supplementary Table S1. Phenological growth stage of *B. distachyon* plants sampled at two-week intervals based on the BBCH scale (Hong *et al.*, 2011).

Supplementary Fig. S1. Basic Fuchsin fluorescence intensity signal is lost primarily from the epidermis following saponification.

Supplementary Fig. S2. Basic Fuchsin is sensitive to the relative amount of lignin between samples but may be insensitive to lignin composition.

Supplementary Fig. S3. Flowering time of *B. distachyon* ecotypes.

Supplementary Fig. S4. Variation in developmental morphology in *B. distachyon* ecotypes.

Supplementary Fig. S5. ABSL changes over developmental time in *B. distachyon* ecotypes.

Supplementary Fig. S6. Basic Fuchsin fluorescence intensity μm^–2^ fluctuates, but generally increases throughout development for *B. distachyon* ecotypes.

Supplementary Data

## Abbreviations:

ABSL: acetyl bromide soluble lignin
AFU: arbitrary fluorescence units
FA: ferulic acid
pCA: p-coumaric acid
ROI: region of interest.
