# Supplementary material for: Imaging with the fluorogenic dye Basic Fuchsin reveals subcellular patterning and ecotype variation of lignification in Brachypodium distachyon
Source: J Exp Bot. 2015 Apr 28;66(14):4295–304. doi: 10.1093/jxb/erv158 (PMC4493785; doi:10.1093/jxb/erv158)
Supplement: Supplementary Data [file supp_66_14_4295__index.html]

Imaging with the fluorogenic dye Basic Fuchsin reveals subcellular patterning and ecotype variation of lignification in Brachypodium distachyon — Imaging with the fluorogenic dye Basic Fuchsin reveals subcellular patterning and ecotype variation of lignification in Brachypodium distachyon — Supplementary Data 

# Imaging with the fluorogenic dye Basic Fuchsin reveals subcellular patterning and ecotype variation of lignification in *Brachypodium distachyon*

## Supplementary Data

Data files

**Files in this Data Supplement:**

- Supplementary Data - Supplementary Data
