# Supplementary material for: Imaging with the fluorogenic dye Basic Fuchsin reveals subcellular patterning and ecotype variation of lignification in Brachypodium distachyon
Source: J Exp Bot. 2015 Apr 28;66(14):4295–304. doi: 10.1093/jxb/erv158 (PMC4493785; doi:10.1093/jxb/erv158)
Supplement: Supplementary Data [file supp_erv158_jexbot139824_file001.pdf]

Imaging with the Fluorogenic Dye Basic Fuchsin Reveals Subcellular Patterning and Ecotype Variation of Lignification in Brachypodium distachyon

Nikki E. Kapp, William J. Barnes, Tom L. Richard, and Charles T. Anderson

Supplemental Files

Table S1. Phenological growth stage of *B. distachyon* plants sampled at two-week intervals based on the BBCH scale (Hong *et al*., 2011). The beginning of heading (stage 51) was used as the earliest classification of flowering plants.

				BBCH growth stage			
	plant #	sampling time: **2**	**4**	**6**	**8**	**10**	**12**
Bd21-3	1	21	59	69	92	92	92
	2	21	53	65	92	92	92
	3	21	59	59	92	87	92
	4	21	59	69	92	92	92
	5	21	59	69	92	92	92
Adi-10	1	21	25	37	37	55	69
	2	21	25	37	37	65	37
	3	21	21	37	37	59	92
	4	21	37	37	37	59	69
	5	21	37	37	37	37	69
Bd1-1	1	21	37	69	37	53	37
	2	21	55	65	37	37	37
	3	21	37	65	69	37	92
	4	21	37	65	37	92	92
	5	21	37	65	87	53	37
**Additional samples for ABSL**							
Bd21-3	1	21	59	69	87	92	92
	2	21	59	69	87	92	92
	3	21	59				
Adi-10	1	21	37	37	37	37	65
	2	21	37	37	37	59	65
	3	21	37				
Bd1-1	1	21	51	69	87	37	92
	2	21	37	69	87	92	92
	3	21	37				

Supplementary Figures

Figure S1. Basic Fuchsin fluorescence intensity signal is lost primarily from the epidermis following saponification. Profile of fluorescence intensity averaged from vascular bundles of a four-week-old Basic Fuchsin-stained Bd21-3 stem section before and after saponification.

Figure S2. Basic Fuchsin is sensitive to the relative amount of lignin between samples but may be insensitive to lignin composition. Average fluorescence intensity (E) of Basic Fuchsin stained *A. thaliana* Col before (A, A') and after (B, B') saponification, and *fah1-2* (C, C') and *ref3-2* (D, D') stem sections. Images labeled with a prime (A' to D') show Basic Fuchsin fluorescence with a pseudo-color look-up table applied where the intensity profile shows pixel intensity values for different colors. Error bars show standard error; different letters indicate significant differences measured by one-way ANOVA and post-hoc test, $p<0.05$.

Figure S3. Flowering time of *B. distachyon* ecotypes. *B. distachyon* ecotype Bd21-3 flowers earlier than Bd1-1 and Adi-10. Heading was scored every three days throughout development for a sub-population of ecotypes Bd21-3, Bd1-1 and Adi-10 (n = 30 plants per ecotype).

Figure S4. Variation in developmental morphology in *B. distachyon* ecotypes. *B. distachyon* ecotype Adi-10 produces more vegetative tissue throughout development than ecotypes Bd21-3 and Bd1-1. Representative images of *B. distachyon* ecotypes Bd21-3, Bd1-1, and Adi-10 sampled every two weeks from two to twelve weeks. Scale bar = 10 cm.

Figure S5. ABSL does not increase significantly over developmental time in *B. distachyon* ecotypes. Error bars represent standard error of five biological replicates with three technical replicates each. Asterisks indicate significant differences between ecotypes at each time point measured by one-way ANOVA and post-hoc test, $p<0.05$. Asterisks indicate significant differences between ecotypes at each timepoint (A), or letters indicated differences within ecotypes across timepoints (B-D) measured by one-way ANOVA and post-hoc test ($p<0.05$).

Figure S6. Basic Fuchsin fluorescence intensity µm-2 fluctuates, but generally increases throughout development for *B. distachyon* ecotypes. Error bars represent standard error of five biological replicates with three technical replicates for each. Asterisks indicate significant differences between ecotypes at each timepoint (A), or letters indicated differences within ecotypes across timepoints (B-D) measured by one-way ANOVA and post-hoc test ($p<0.05$).

Figure S1

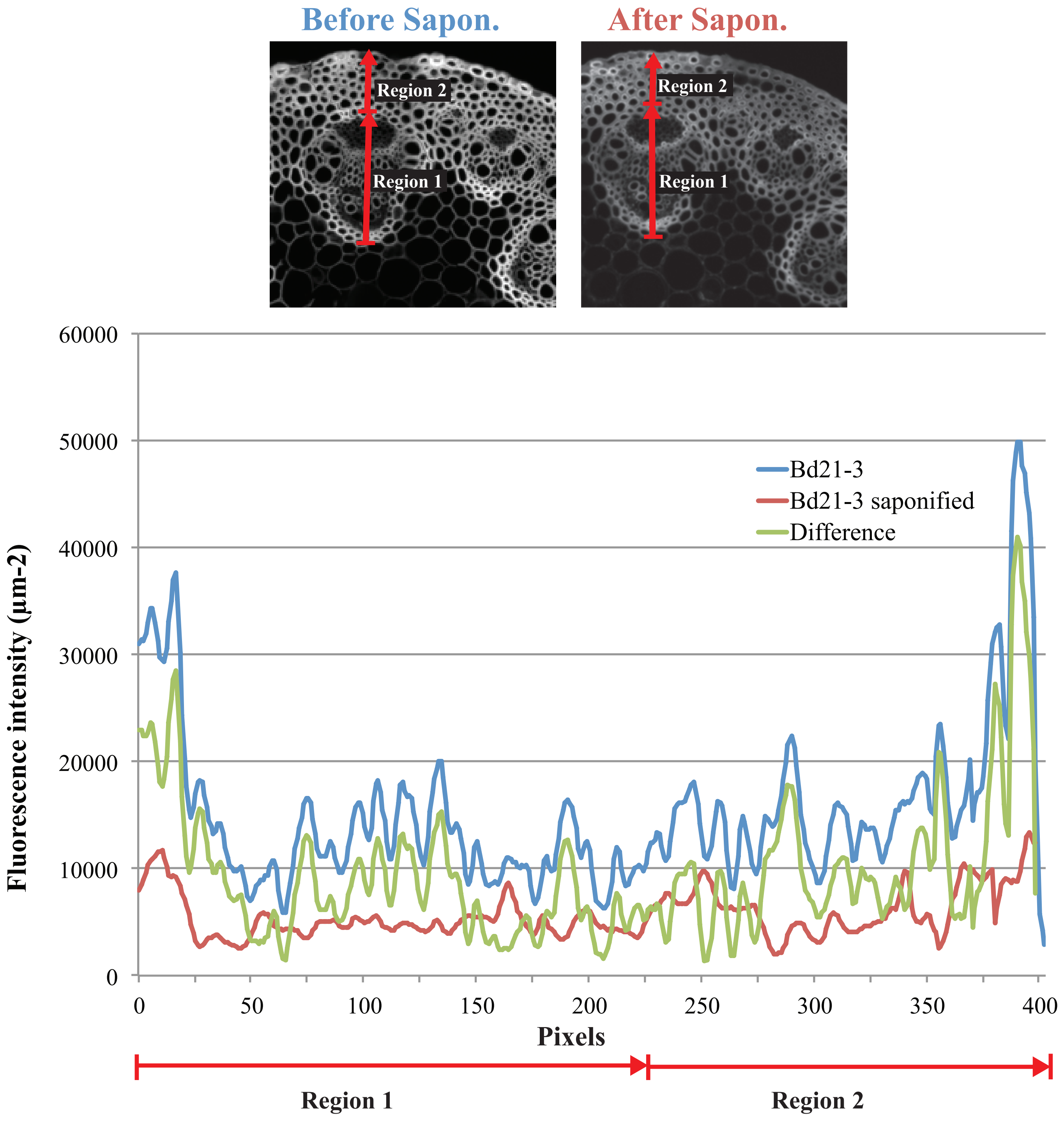

Before Sapon.
After Sapon.
Region 2
Region 1
Region 2
Region 1
60000
50000
40000
30000
20000
10000
0
Fluorescence intensity (µm-2)
Bd21-3
Bd21-3 saponified
Difference
0
50
100
150
200
250
300
350
400
Pixels
Region 1
Region 2

Figure S2

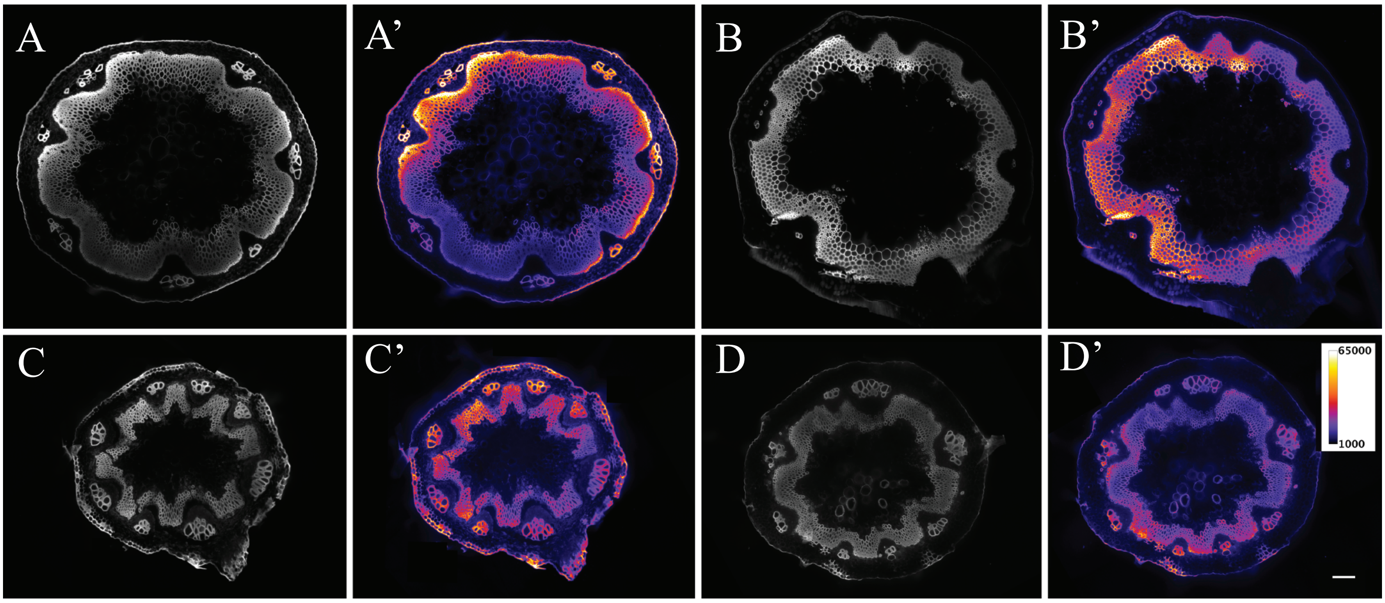
A
A'
B
B'
C
C'
D
D'
65000
1000

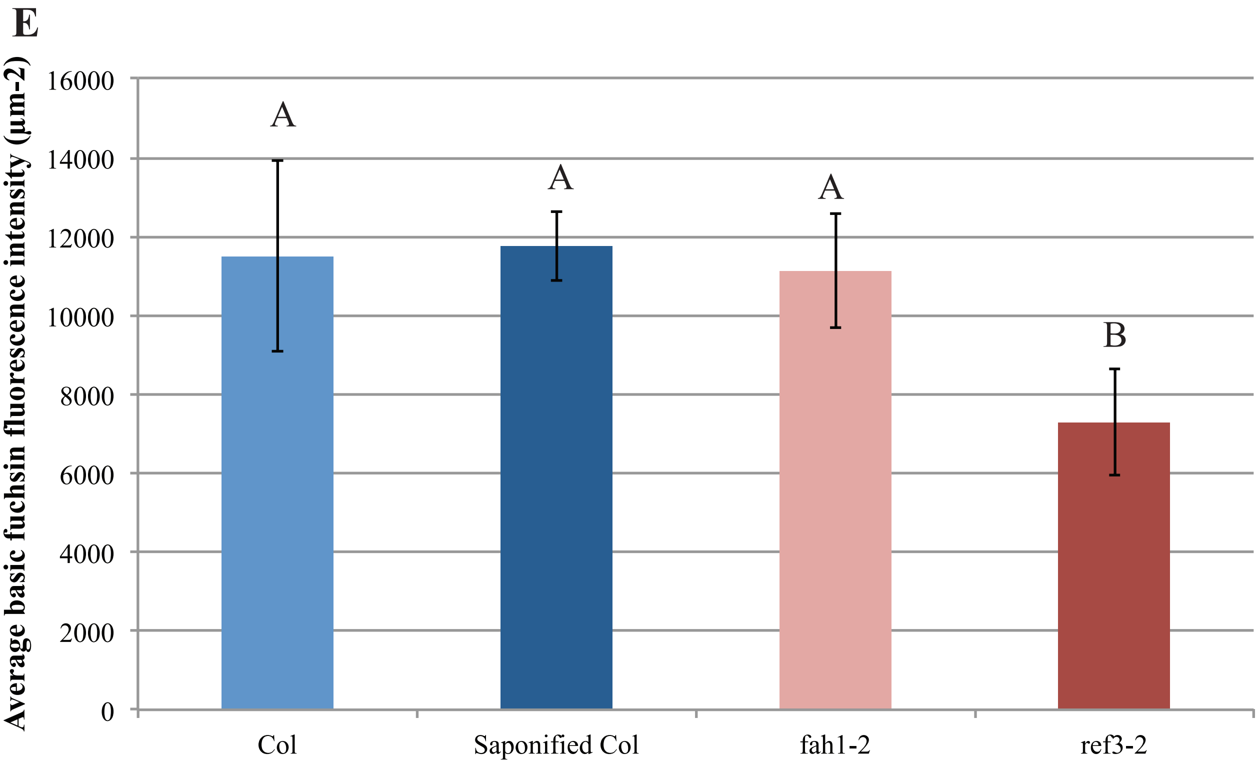
E
Average basic fuchsin fluorescence intensity (µm-2)
16000
14000
12000
10000
8000
6000
4000
2000
0
A
A
A
B
Col
Saponified Col
fah1-2
ref3-2

Figure S3

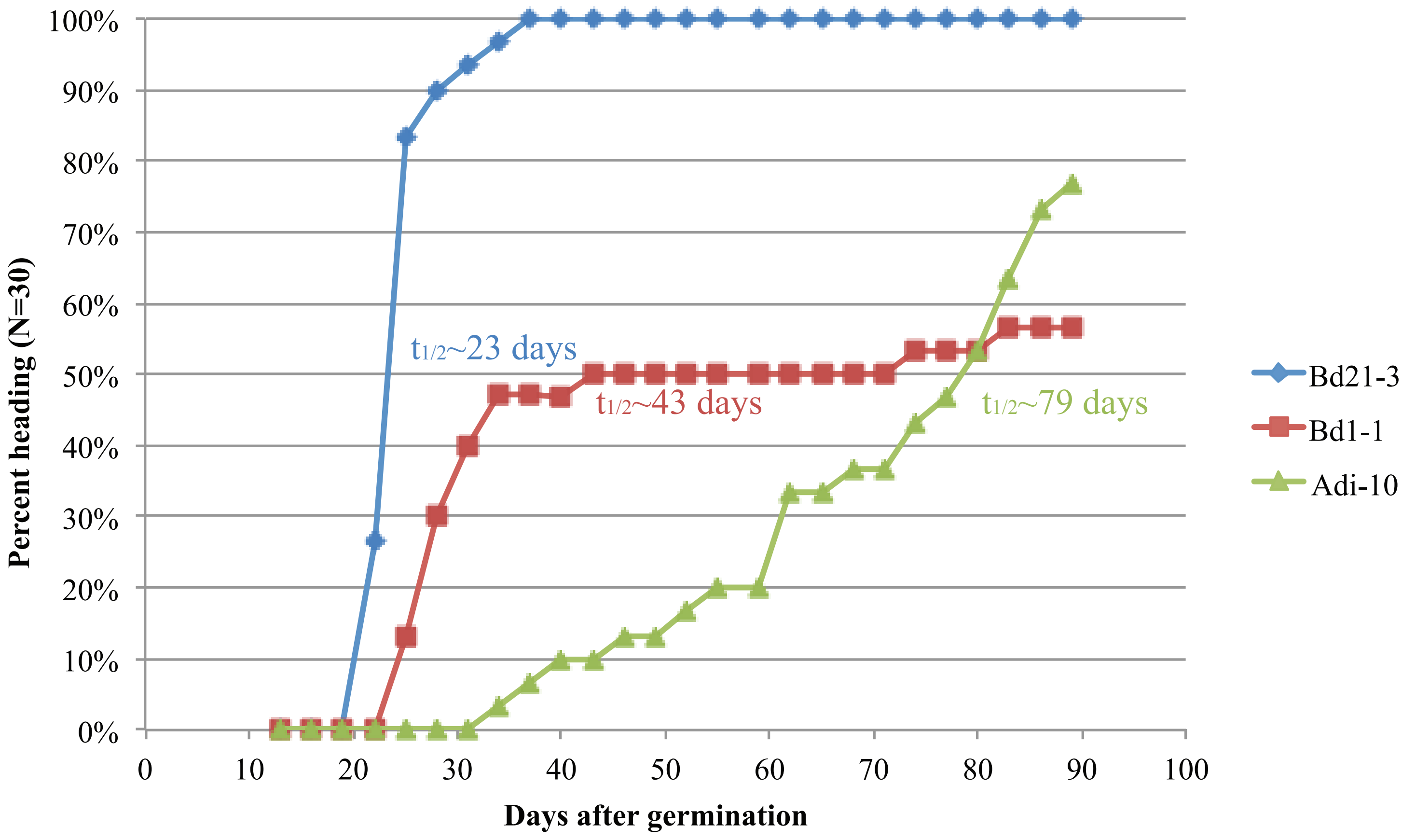

100%
90%
80%
70%
60%
50%
40%
30%
20%
10%
0%
Percent heading (N=30)
0
10
20
30
40
50
60
70
80
90
100
Days after germination
t1/2~23 days
t1/2~43 days
t1/2~79 days
Bd21-3
Bd1-1
Adi-10

Figure S4

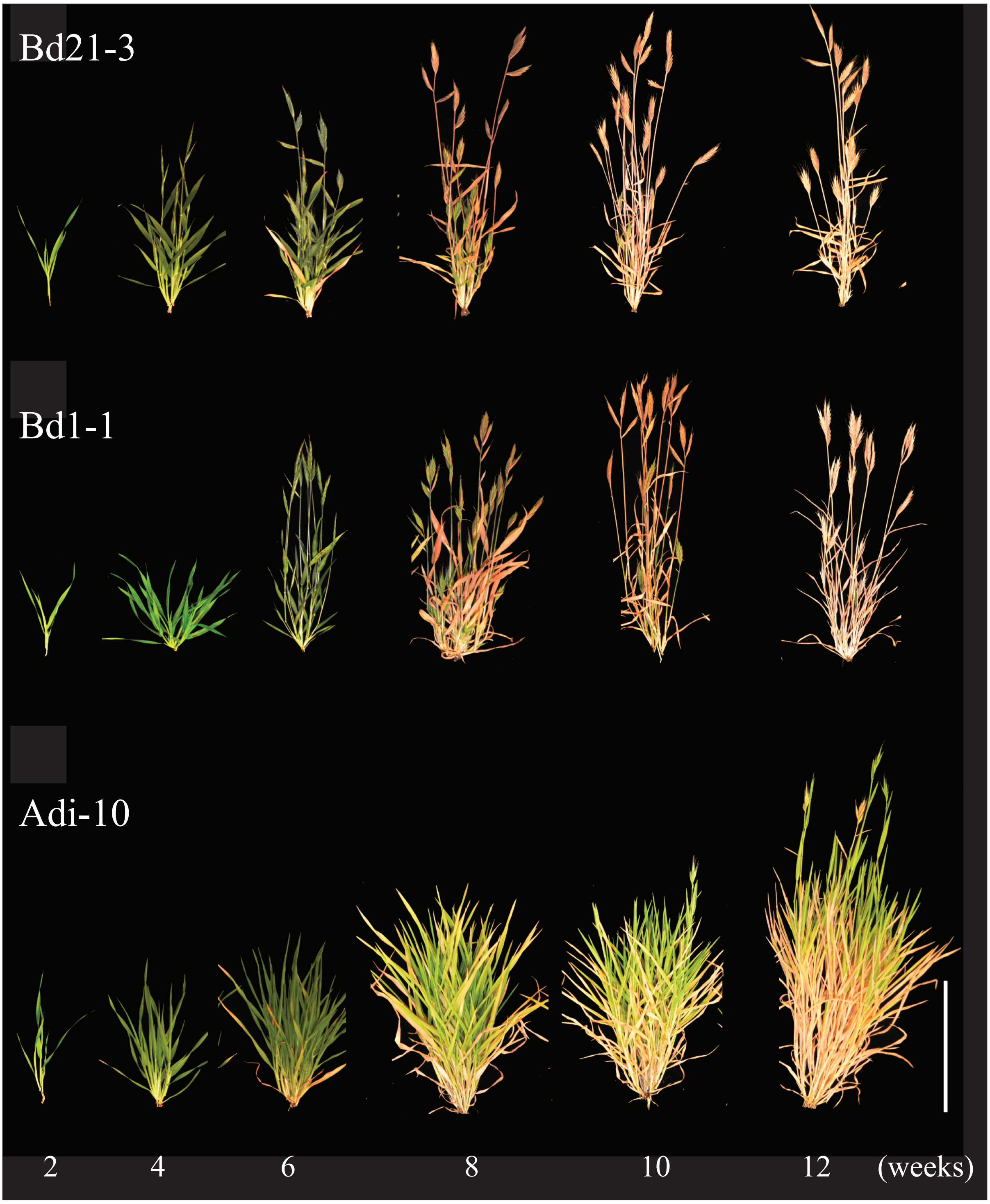

Figure S5

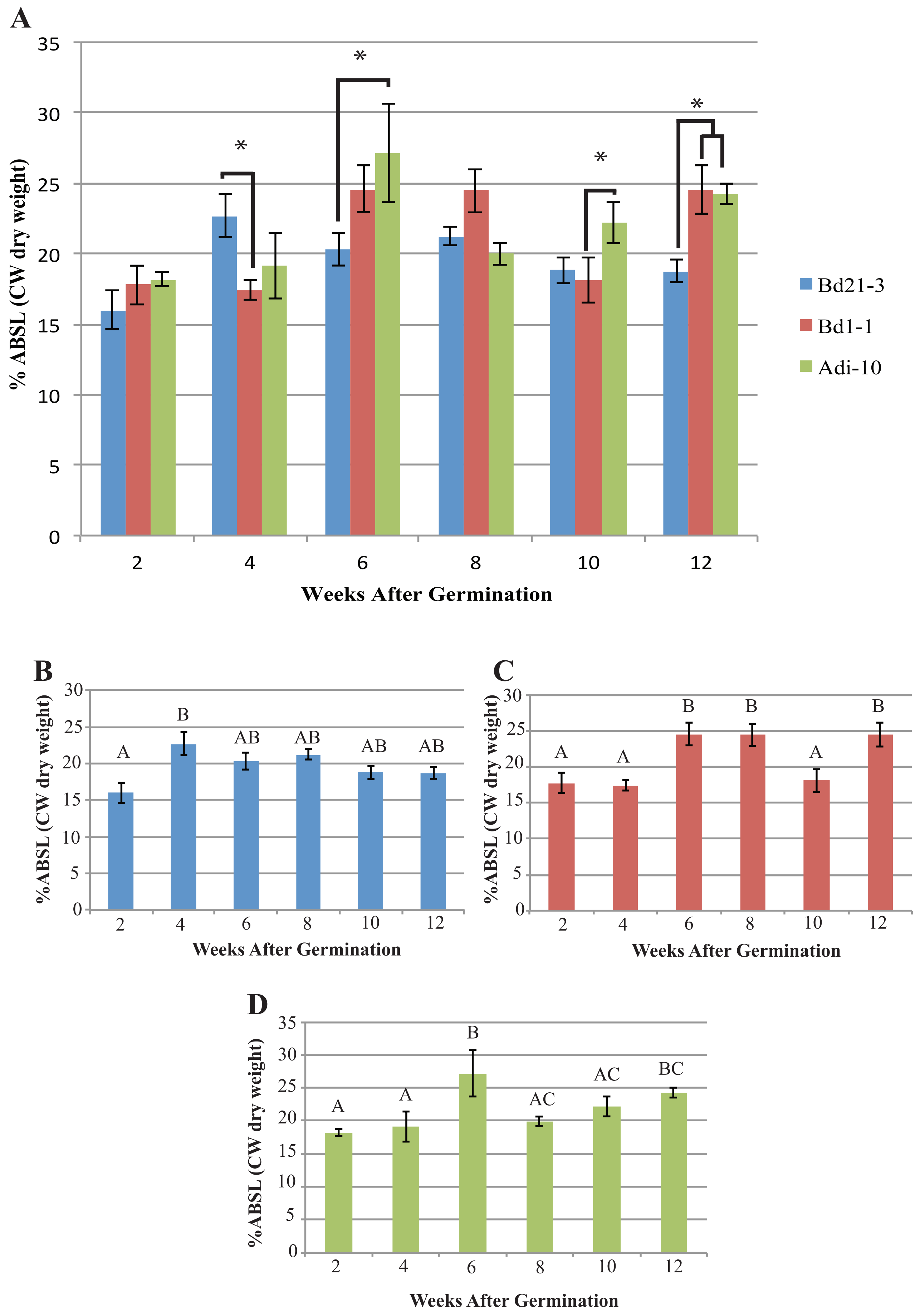

A
% ABSL (CW dry weight)
Weeks After Germination
Bd21-3
Bd1-1
Adi-10
B
%ABSL (CW dry weight)
Weeks After Germination
C
%ABSL (CW dry weight)
Weeks After Germination
D
%ABSL (CW dry weight)
Weeks After Germination

Figure S6

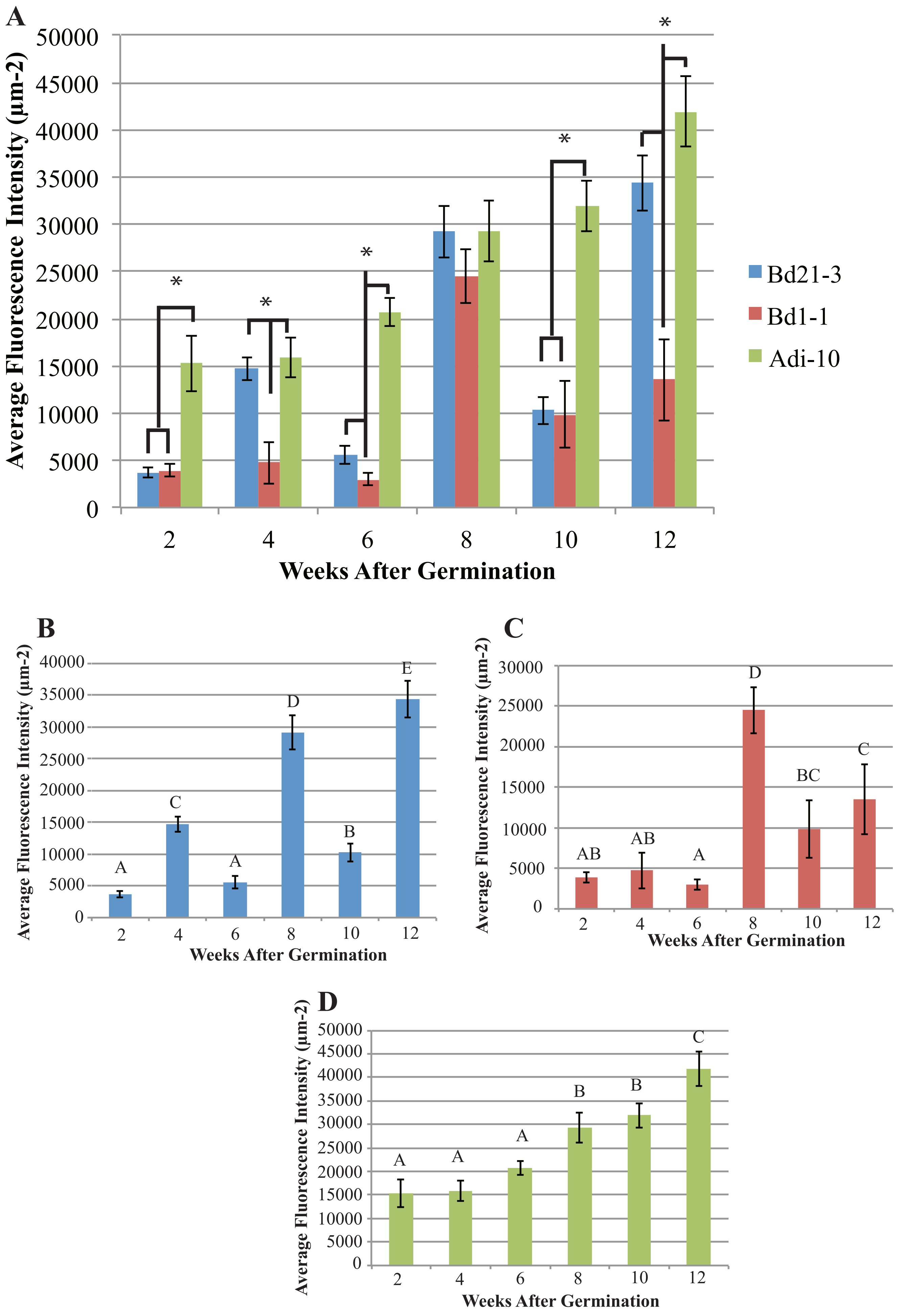
A
B
C
D
Average Fluorescence Intensity (μm-2)
Weeks After Germination
Bd21-3
Bd1-1
Adi-10
*
A
C
A
D
B
E
AB
AB
A
D
BC
C
A
A
A
B
B
C
